# The Role of the Immune System in Nevirapine-Induced Subclinical Liver Injury of a Rat Model

**DOI:** 10.5402/2012/932542

**Published:** 2012-08-16

**Authors:** Zanelle Bekker, Andrew Walubo, Jan B. du Plessis

**Affiliations:** Department of Pharmacology, University of the Free State, P.O. Box 339 (G6), Bloemfontein 9300, South Africa

## Abstract

In this study, the role of the immune system in nevirapine- (NVP-) induced subclinical liver injury was investigated by observing for changes of some immune parameters during the initial stages of NVP-induced hepatotoxicity in a rat model. In the acute phase, two test-groups of 10 Sprague-Dawley rats each were administered with bacterial lipopolysaccharide (LPS) or saline (S) intraperitoneally, followed by oral NVP, after which 5 rats from each group were sacrificed at 6 and 24 hours. For the chronic phase, two groups of 15 rats each received daily NVP, and on days 7, 14, and 21, five rats from each group were administered with either LPS or S, followed by that day's NVP dose, and were sacrificed 24 hours later. NVP caused liver injury up to seven days and progressively increased IL-2 and IFN-**γ** levels and lymphocyte count over the 21 days. NVP-induced liver injury was characterized by apoptosis and degeneration changes, while, for LPS, it was cell swelling, leukostasis, and portal inflammation. Coadministration of NVP and LPS attenuated NVP-induced liver injury. In conclusion, the immune system is involved in NVP toxicity, and the LPS effects may lay the clue to development of therapeutic strategies against NVP-induced hepatotoxicity.

## 1. Introduction

Nevirapine (NVP) is a nonnucleoside reverse transcriptase inhibitor (NNRTI) used for the prophylaxis and treatment of human immunodeficiency virus (HIV) infections. Unfortunately, NVP is associated with severe skin and hepatic hypersensitivity reactions that have hampered its use particularly for HIV prophylaxis [1]. The hepatotoxicity is common in patients with higher CD4 counts and in the first three weeks of NVP treatment [2, 3]. Whereas the mechanism of NVP-induced hepatotoxicity remains unknown, it was postulated to be immune mediated [4, 5]. Such an association has already been proven in animal models for NVP-induced skin reactions [6–8]. Likewise, several drugs have been shown to induce hepatotoxicity in association with an activated immune system, that is, diclofenac [9], paracetamol [10–13], bacterial lipopolysaccharide (LPS) plus ranitidine [14], and trovafloxacin [15].

The immune pathways that consequently cause the liver damage have been likened to the pathogenesis of liver injury in diseases such as hepatitis B virus (HBV) and hepatitis C virus (HCV) infections where an activated cell-mediated immunity was incriminated for the liver damage [16, 17]. This is evidenced by a rise in the type 1 T-helper cells (Th_1_) proinflammatory cytokines interleukin 2 (IL-2), interferon-gamma (IFN-*γ*), and tumor necrosis factor-alpha (TNF-*α*), with reduced or failing type 2 T-helper cells (Th_2_) anti-inflammatory cytokines interleukin 4 (IL-4), interleukin 5 (IL-5), interleukin 6 (IL-6), and interleukin 10 (IL-10) [18]. In fact, HBV and HCV are proven risk factors for NVP-induced hepatotoxicity [2, 3]. This and the fact that NVP-induced hepatotoxicity is common in patients with higher CD4 counts imply that increased stimulation of the cell-mediated immune response in some HIV-positive patients may predispose them to NVP-induced hepatotoxicity. However, the observation that it takes some weeks to develop liver injury means that NVP itself plays a role in the initiation of the lesion. Here, it was envisaged that NVP activates the cell-mediated immune response, leading to liver injury that is propagated by the drug itself or the immune system. As such, a study on the role of the immune system in NVP-induced subclinical liver injury was undertaken with a hope that it will shed light on the mechanism and possible modes of therapy for NVP toxicity. The subclinical liver injury was necessary to ensure that the immune changes would not be complicated or distorted by the extensive necrosis associated with overt hepatotoxicity.

## 2. Materials and Methods

### 2.1. Materials and Reagents

NVP oral suspension (50 mg/5 mL) and tablets (Viramune) 200 mg (Boehringer Ingelheim Pharmaceuticals, Inc., Ridgefield, CT, USA), sterile normal saline (Euro-Med Laboratories Inc., Cavite, Philippines) were purchased from a local pharmacy, while bacterial lipopolysaccharide (*Escherichia coli*) was from Sigma-Aldrich (St. Louis, MO, USA). The enzyme-linked immunosorbent assay (ELISA) kit for IL-2 was from Pierce Biotechnology Inc. (Rockford, IL, USA), while the IFN-*γ*  ELISA kit was from Bender MedSystems (Vienna, Austria), and that for TNF-*α*  was from eBioscience (San Diego, CA, USA).

### 2.2. Animal Care

Ethical approval was obtained from the Animal Ethics Committee of the University of the Free State. Sprague-Dawley (SD) rats weighing 260–400 g were used. Animals were kept and treated at the Animal House of the University of the Free State where they were cared for by qualified staff according to International animal care guidelines. They were fed on standard rat chow and water *ad libitum*, and the cages were cleaned twice a week. During treatment, animals were inspected for skin lesions and other visible adverse events every day.

### 2.3. Experimental Design

The experiment was divided in two phases, acute and chronic. The acute phase involved evaluation of animals over 24 hours after single-dose administration of the drugs, while the chronic phase involved evaluation of animals over 21 days during which the drugs were being administered. Of note, the term “chronic” was selected for convenience without implications on the way it is used in preclinical drug-animal testing.

In preliminary experiments, it was found that the rats metabolize NVP very fast such that at doses of 25 and 50 mg/kg/day no NVP was detected in plasma by 24 hours. Ultimately, a dose of 200 mg/kg/day of NVP by oral gavage was selected. The dose of LPS was 2.9 × 10^6^ E.U./kg (E.U.: endotoxin units) intraperitoneally and was based on that used earlier [9]. NVP was given orally and within 5 minutes after administration of LPS or saline because by the time the absorption is complete (1-2 hrs), the immune system would be activated by LPS. In previous studies with diclofenac [9] and travofloxacin [15], where the drugs and LPS were given intravenously, the drugs were administered 2 and 3 hours, respectively, after LPS.

#### 2.3.1. Acute Phase

Animals were divided into three groups of 10 rats each, and drugs were administered to groups as follows: saline and NVP (S + NVP), LPS and Saline (LPS + S), and LPS and NVP (LPS + NVP). From each group, five rats were sacrificed at 6 and 24 hours after drug administration. Another group of 5 animals was not treated as it formed the base line data.

#### 2.3.2. Chronic Phase

Animals were divided into three groups of 15 rats each, and, in two groups, NVP was administered daily to both groups, while only saline was administered instead of NVP to the third group. Then, on days 7, 14, and 21, five rats from each of the two NVP treatment groups were administered intraperitoneally with either LPS or saline followed by that day's NVP dose within 5 minutes, hence LPS + NVP group and S + NVP groups, respectively, while the saline group was administered with saline, hence, S + S group. Thereafter, the animals were sacrificed after 24 hours.

### 2.4. Surgical Procedure and Blood Collection

Under ether anaesthesia, blood (8–10 mL) was drawn via cardiac puncture and immediately aliquoted into the appropriate test-tubes. Thereafter, the abdomen was opened by a vertical incision and the liver was exposed. A liver sample (5–10 g) was cut and stored in 10% formalin and sent for histopathology. The remainder of the liver was excised, removed, washed in a 1.5% potassium chloride solution, frozen with liquid nitrogen, and stored at −85°C. The rat was sacrificed by exsanguination whilst still under anaesthesia. Blood was collected for liver function tests and full blood count (for 21 days group), NVP concentration, and cytokine analysis.

Liver functions tests and full blood count (FBC) were done by Pathcare Veterinary Laboratory (Bloemfontein, South Africa), while NVP concentrations were measured by HPLC using a method developed in our laboratory. Serum cytokines, IL-2, IFN-*γ*, and TNF-*α*, were measured in our laboratory by ELISA using a Multiskan Ascent UV-spectrophotometer with a 96 well microplate reader (Thermo Electric Corp., Shanghai, China). All assays were performed according to the manufacturer's instruction. The liver histopathology studies were done by an independent veterinary pathologist (Golden Vetpath Idex Laboratories, Johannesburg, South Africa). Subclinical liver injury referred to any changes in the liver histology ± changes in liver enzymes (AST, ALT, and ALKP) that were considered pathological, that is, not observed in the normal rat liver.

### 2.5. Data Analysis

Data was analysed by nonparametric methods using the GraphPad Instat statistical program and the Mann-Whitney test was used for data comparison with level of significance set at *P* < 0.05. 

## 3. Results

### 3.1. Direct Observations

On the day of sacrifice, that is, 24 hours after administration of LPS or saline, the animals administered with LPS exhibited goose flesh (raised hair), and, on inspection of the liver during surgery, there were goose bumps on their liver surface. Although, temperature was not measured, this was interpreted as due to an immune reaction akin to serum-sickness.

### 3.2. Acute Phase

**Figure 1:**
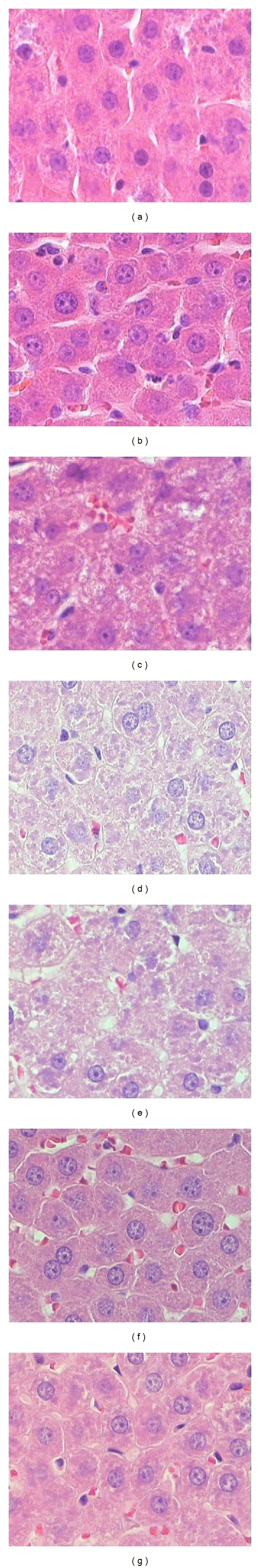
Representative histopathology slides and corresponding pathology reports on rat livers during the acute phase experiment (magnification ×40). (a) Section of rat liver from saline-treated group (Normal liver). (b) Section of rat liver from a group treated with LPS and S (LPS + S) at *6 hours* after dosing (the parenchymal cells are homogenously swollen/hypertrophic with narrow sinusoids, minimal intrahepatic leukostasis, and mild inflammation of the portal tracts). (c) Section of a rat liver from a group treated with LPS and S (LPS + S) at *24 hours* after dosing (the parenchymal cells revealed diffuse vacuolar changes and irregular swollen cytoplasm with prominent encroachment of the sinusoids due to the cell swelling, apoptosis of the hepatocytes and mild portal inflammation). (d) Section of a rat liver from a group treated with S and NVP (S + NVP) at *6 hours* after dosing (mild cloudy swelling and degenerative changes with granular appearance of the cytoplasm in the hepatocytes, apoptosis with diffuse mild hepatocellular swelling; no inflammation). (e) Section of a rat liver from a group treated with S and NVP (S + NVP) at *24 hours* after dosing (mild hepatocellular vacuolar degeneration, apoptosis and dissociated liver parenchymal cells. No sign of inflammation). (f) Section of a rat liver from a group treated with LPS and NVP (LPS + NVP) at *6 hours* after dosing (Normal liver). (g) Section of a rat liver from a group treated with LPS and NVP (LPS + NVP) at *24 hours* after dosing (Normal liver).

Table 1 shows the weights and liver function tests for the four groups of animals in the acute phase. In all animals, there was no change in the liver enzymes suggestive of hepatotoxicity. However, compared to the untreated group (Figure 1(a)), the NVP-treated group (S + NVP) exhibited abnormal histology changes indicative of subclinical liver injury (Figure 1(d)). This subclinical liver injury was characterized, at 6 hours, by mild cloudy swelling and degenerative changes with granular appearance of hepatocytes, increased apoptosis, and diffuse mild hepatocellular swelling (Figure 1(d)), while at 24 hours, it was mainly hepatocellular vacuolar degeneration, apoptosis, and dissociated liver parenchymal cells (Figure 1(e)). The LPS-treated group (LPS + S) too exhibited abnormal histology at 6 and 24 hours, but this was characterized by diffuse vacuolar changes and swollen cytoplasm leading to narrow sinusoids (Figures 1(b) and 1(c)). The pathology implies that the mechanisms of LPS and NVP-induced subclinical liver injury may be different, whereby the pathology of the LPS-induced subclinical liver injury was more generalized while that of NVP was intracellular and affected the hepatocytes. Surprisingly, the group treated by a combination of LPS and NVP (LPS + NVP) exhibited normal histology at 6 and 24 hours, implying that coadministration of LPS and NVP prevented the abnormal histology changes observed with either drug.

**Figure 2:**
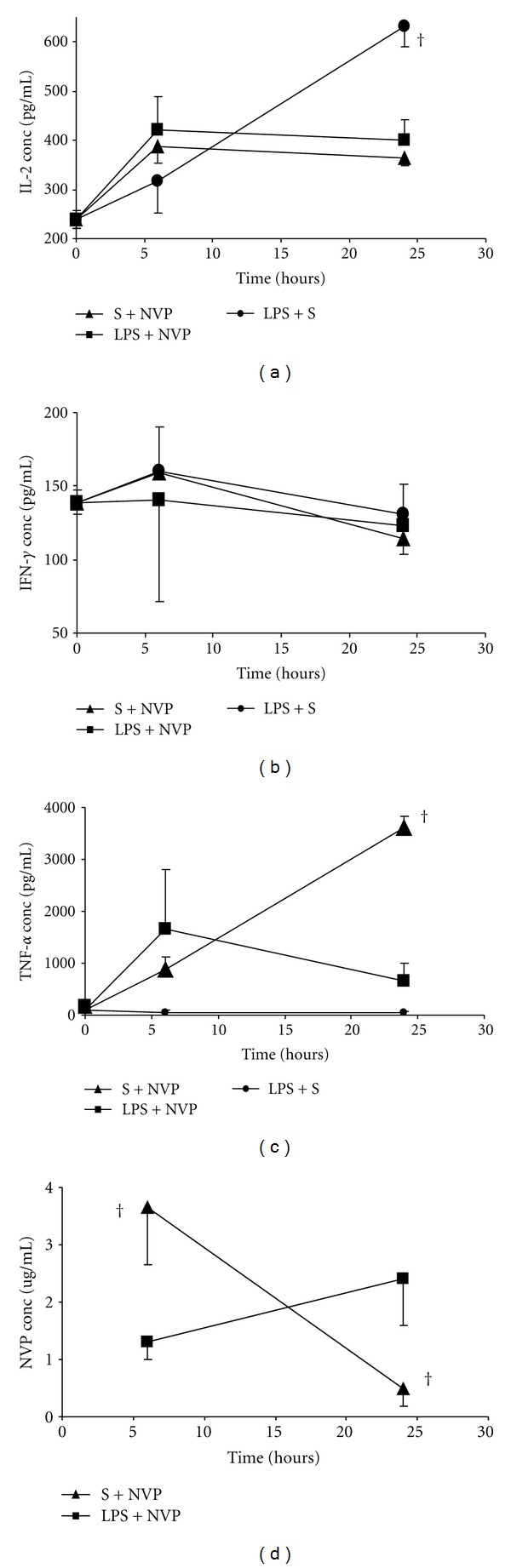
Serum concentration (mean ± SD) of IL-2 (a), IFN-*γ*  (b), TNF-*α*  (c), and NVP (d) at 6 and 24 hours after dosing with saline (S) and nevirapine (NVP: S + NVP); lipopolysaccharide (LPS) and NVP (LPS + NVP); LPS and S (LPS + S). Note^†^: indicates that the values for the nevirapine-treated (S + NVP) group were significantly (*P* < 0.05) different from those of the LPS + NVP treated group.

The changes in cytokine levels are shown in Figure 2. Administration of LPS alone caused a marked increase in IL-2 levels which, by 24 hours, was significantly different (*P* = 0.0286) from that after administration of NVP alone, as well as coadministration of the two drugs (*P* = 0.0159) (Figure 2(a)). There was no change in IFN-*γ*  levels (Figure 2(b)) but NVP lead to increased TNF-*α*  concentrations such that by 24 hours they were significantly different (*P* = 0.0357) from those after LPS administration as well as in the group where NVP and LPS were coadministered (*P* = 0.0357) (Figure 2(c)).  Figure 2(d) shows that by 6 hours NVP concentrations, though not statistically different (*P* = 0.1508) from either group, were lower in the group coadministered with NVP and LPS (NVP + LPS) than in the NVP-treated group (S + NVP), but by 24 hours, this had reversed whereby NVP plasma concentrations in the LPS + NVP group were significantly (*P* = 0.0079) higher than in the S + NVP group.

### 3.3. Chronic Phase

Table 2 shows the weights and liver function tests for animals during the chronic phase. There were more changes in weight in the LPS + NVP group than in the NVP-treated group but because the changes were 5%–10% of body weight, they were attributed to mild-moderate dehydration due to loss of appetite caused by LPS-induced immune sickness, as indicated under the direct observations. Again, there were no changes in liver enzymes suggestive of hepatotoxicity. However, as it was in the acute phase, there were histopathology changes in the livers of some animals suggestive of subclinical liver injury.

**Figure 3:**
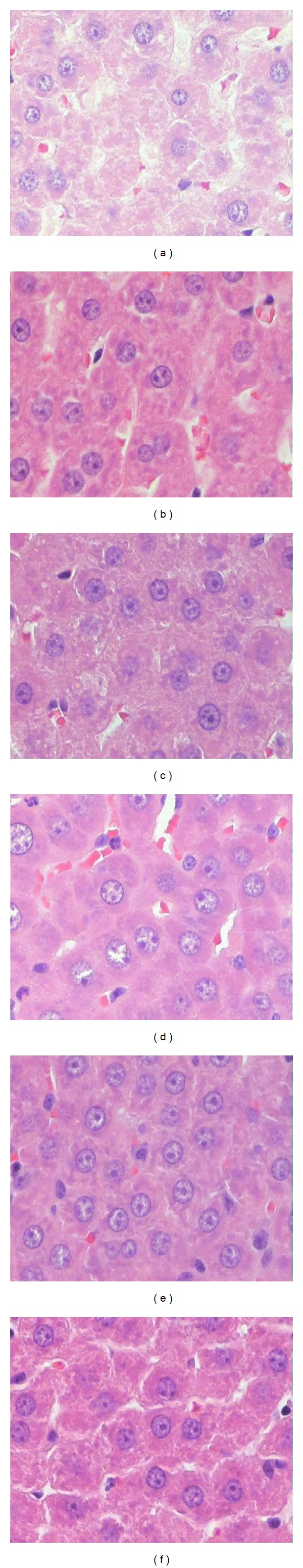
Representative histopathology slides and corresponding pathology reports on a rat liver during the chronic phase (magnification ×40). (a) Section of a rat liver 24 hours after dosing with S and NVP in a group treated daily with NVP for *7 days *(S + NVP). Pathology report: “*mild centrilobular hepatocellular degeneration and cell swelling with a cloudy appearance of the cytoplasm of hepatocytes in the central part of the liver lobules. On the periphery, moderate hepatocellular apoptosis. The Kuppfer cells are prominent with mild lymphocytic infiltration of the portal tracts and isolated perivascular lymphoplasmacytic cuffing *…”. (b) Section of a rat liver 24 hours after dosing with S and NVP in a group treated daily with NVP for *14 days* (S + NVP). Pathology report: “*Normal liver”. *(c) Section of a rat liver 24 hours after dosing with S and NVP in a group treated daily with NVP for *21 days* (S + NVP). Pathology report: “*Normal liver”*. (d) Section of a rat liver 24 hours after dosing with LPS and NVP in a group treated daily with NVP for *7 days* (LPS + NVP). Pathology report: “*moderate apoptosis, and mild lymphocytic infiltration with perivascular distribution in the portal areas.” *(e) Section of a rat liver 24 hours after dosing with LPS and NVP in a group treated daily with NVP for *14 days* (LPS + NVP). Pathology report: “*Normal liver”. *(f) Section of a rat liver 24 hours after dosing with LPS and NVP in a group treated daily with NVP for *21 days* (LPS + NVP). Pathology report: “*Normal liver.” *

Figure 3 shows the representative slides and reports of the liver histopathology. The group administered NVP only (S + NVP) exhibited abnormal liver histology on day 7 that was characterized by centrilobular hepatocellular degeneration and cell swelling with a cloudy appearance of the cytoplasm of hepatocytes, hepatocellular apoptosis, and prominent perivascular lymphoplasmacytic cuffing (Figure 3(a)). However, this liver injury dissipated with continual administration of NVP such that by days 14 and 21, there was no evidence of subclinical liver injury (Figures 3(b) and 3(c)). On the other hand, the group coadministered with LPS and NVP (LPS + NVP) exhibited a normal histology on all occasions, that is, days 7, 14, and 21, again, illustrating further that LPS attenuates the NVP-induced subclinical liver injury, as it was in the acute phase (Figures 3(d)–3(f)).

Table 3 shows the full blood count (FBC) data after 21 days of treatment. The group coadministered with LPS and NVP exhibited a leukocytosis (15.33 ± 2.1^9^/L) that was characterized by a high neutrophil count (8.89 ± 1.3^9^/L) with moderate lymphocytosis (5.33 ± 1^9^/L), while the group administered with NVP exhibited marked lymphocytosis (7.95 ± 1.4^9^/L) with no effect on other white cells. Interestingly, on chronic therapy, both groups exhibited marked thrombocytosis (increased platelet count) on day 21, that is,  975 ± 184^9^/L  in the NVP-treated group and 601 ± 85.4^9^/L in the group coadministered with LPS and NVP.

**Figure 4:**
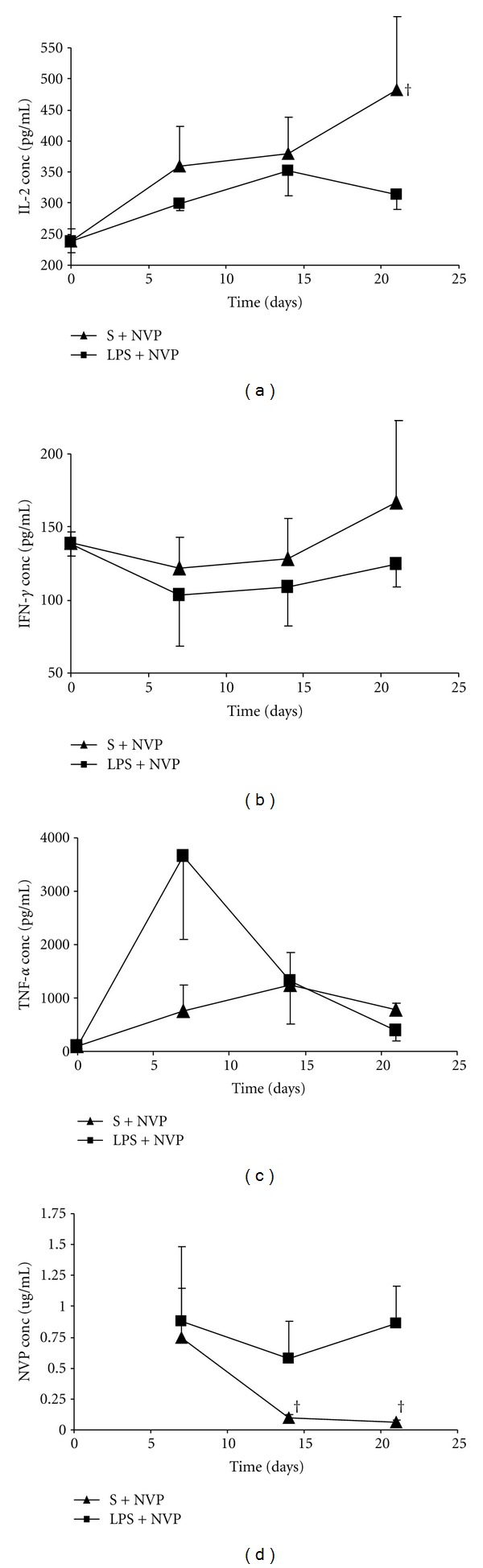
Serum concentrations (mean ± SD) of IL-2 (a), IFN-*γ*  (b), TNF-*α*  (c) and nevirapine (NVP), (d), in rats treated daily with NVP, but 24 hours after challenge with saline (S; S + NVP) or lipopolysaccharide (LPS; LPS + NVP) on days 7, 14 and 21 treatment. Note^†^: indicates that the values for the nevirapine-treated group (S + NVP) were significantly (*P* < 0.05) different from those of the LPS + NVP group.

Regarding the changes in cytokine levels on chronic administration, the group administered with NVP exhibited a progressive increase in IL-2 concentrations such that day 21, the levels were significantly (*P* = 0.0357) different from those in the group coadministered with LPS and NVP (Figure 4(a)). Likewise, the concentrations of IFN-*γ*  were increased but they were not different from either group (Figure 4(b)). Whereas TNF-*α*  exhibited increased concentration on day 7 in the group coadministered with LPS and NVP, the subsequent concentration on days 14 and 21 were not different from either group (Figure 4(c)).  Figure 4(d) shows that NVP plasma concentrations were higher in the group coadministered with LPS and NVP than in the NVP only group on days 14 (*P* = 0.0286) and 21 (*P* = 0.0357) of treatment.

## 4. Discussion

This study presents an animal model by which to undertake further evaluation of some of the clinical and epidemiological observations on NVP-induced hepatotoxicity, with a hope to elucidate on the mechanism of NVP immune-mediated hepatotoxicity and search for potential new therapeutic strategies [19, 20]. Of note, subclinical liver injury was used here to imply potential for progression to hepatotoxicity. Hepatotoxicity is defined as “a three-fold or more raise in liver enzymes,” and in many instances this is also referred to as “clinical hepatotoxicity.” However, from our previous report on NVP-induced hepatotoxicity, it was shown that although the plasma levels of liver enzymes for the rats pre-treated with NVP were not significantly different from the control group, indicating no hepatotoxicity, the histological findings in the NVP-treated rats depicted liver injury [21]. Also, because similar changes were observed, albeit being more severe, in the histology of animals with overt hepatotoxicity, it was concluded that this was indeed a subclinical stage of hepatotoxicity or liver injury. Accordingly, by using these early histopathological changes in the liver as the prodromal symptoms of clinical hepatotoxicity, the current study was able to demonstrate the possible changes in some immune markers during NVP therapy at a stage when the cellular and/or immune regulatory mechanisms are still intact. Of note, in the same report by Walubo et al. [21], clinical hepatotoxicity was associated with extensive cell damage or necrosis of hepatocytes, biliary system, and connective tissue, which would complicate interpretation of observations in this study. Therefore, for this study, using the said animal model of NVP-induced hepatotoxicity, changes in serum levels of some of the well-characterized Th1 cytokines (IL-2, IFN-*γ*, and TNF-*α*) during NVP-induced subclinical hepatotoxicity were investigated to determine the role immune system in NVP-induced subclinical hepatotoxicity.

NVP caused liver injury within hours after the first dose and this continued up to 7 days. However, by days 14 and 21, there was no evidence of subclinical liver injury, which implied that with time, the body overcame the pathological process of NVP-induced subclinical liver injury. This could be partly explained by recent reports which showed that NVP was metabolically activated to quinone methidine, a toxic reactive metabolite, by CYP3A4, and, to a lesser extent, CYP2D6, CYP2C19, and CYP2A6 [22], and that this reactive metabolite was responsible for NVP-induced skin reactions [23]. Although not yet proven *in vivo*, the toxicity was postulated to be via formation of antigenic protein adducts by the quinone methidine, and some of the NVP-protein adducts have been synthesized *in vitro* [24]. It was also shown that although the quinone methidine is normally eliminated by glutathione conjugation, it is a mechanism-based inactivator of CYP3A4 [22], implying that, with time, this metabolite may inhibit its formation, and this may partly explain the amelioration of liver injury after 14 and 21 days of treatment.

Surprisingly, coadministration of LPS and NVP prevented the liver injury by either drug by yet unexplained mechanism. However, LPS is known to induce release of several cytokines that inhibit CYP450 [25–28]. Administration LPS to rats inhibited protein and mRNA expression for CYP2C8, CYP2C19, and CY3A4 [25–28], as such, it is plausible to postulate that LPS lead to inhibition of the metabolic activation of NVP. This was supported by the high concentration of NVP in animals treated with LPS + NVP. Another mechanism is that LPS activated the innate immunity which could have led to effective elimination of the antigenic products, leading to early immune tolerance [16]. As the two mechanisms involve the immune system, this suggests that manipulation of the immune system has the potential to prevent NVP-induced liver injury, and that understanding the mechanism involved may give a clue to development of an ultimate therapeutic agent. Conversely, the mechanism by which NVP ameliorated the LPS-induced liver injury was not clear but it could be due to competitive interference by NVP immunogenic metabolites. Nevertheless, whether this effect is mediated through the immune system or not, it could not be deduced from the data in this study.

Most important was that both LPS and NVP led to stimulation of the immune system as evidenced by increased Th1 cytokines (IL-2, IFN-*γ*, and TNF-*α*). These cytokines activate macrophages and promote cell-mediated immune responses against invasive intracellular pathogens, enhancing fever, and tissue destruction [29]. TNF-*α*  promotes inflammation and apoptosis, while IL-2 increases the killing ability of natural killer (NK) cells and synthesis of other cytokines, including IFN-*γ*  [30]. Furthermore, together with TNF-*α*, IL-2 controls the induction of both Th1 and Th-2 responses by promoting T cell division and antibody synthesis by B cells [18]. IFN-*γ*  is produced by NK and T cells. It has antiviral activities as it activates the pathway that leads to induction of Cytotoxic T-cells and augments TNF activity. It induces nitric oxide (NO) by NO synthetase which mediates apoptosis of damaged cells and killing of bacteria by macrophages [29, 31]. In effect, the three cytokines (IL-2, IFN-*γ*, and TNF-*α*) mediate the destructive process of the body's defense mechanism (innate immunity), and both NVP and LPS, independent of each other, led to stimulation of this defense mechanism. Whereas for the LPS, it was a necessary defense against bacterial infection, it is not clear why (and how) NVP stimulated this defense mechanism, but it could be part of the “immune tolerance” mechanisms in the liver.

Since continual stimulation of the immune system (IL-2, IFN-*γ*, and TNF-*α*) by NVP was not associated with progression of NVP-induced liver injury, particularly after 7 days, it seems to dispel claims that the immune system has a role in NVP-induced liver injury. However, a recent report on the metabolic activation of NVP to toxic metabolites in humans, and the current observation of differences in the NVP concentrations between the NVP only group and LPS + NVP group suggest otherwise [32]. They suggest that a similar phenomenon to acetaminophen induced hepatotoxicity [11]: that NVP was metabolized to reactive metabolites which were converted to immunogenic protein adducts, and these then triggered a cell-mediated adaptive immune response, leading to initiation of the destruction or programmed elimination of hepatocytes (increased apoptosis) expressing the metabolic adducts. As indicated earlier, NVP protein adducts have been synthesized *in vitro* [24]. Accordingly, in this study, the lower concentrations of NVP in the NVP only group implied that more NVP was metabolized to the immunogenic metabolites leading to increased immune stimulation and liver injury, while the higher NVP concentrations in LPS + NVP group implied that less NVP was metabolized to the immunogenic metabolites, and therefore little or no liver injury occurred. In effect, LPS attenuated NVP-induced liver injury by inhibiting the metabolism of NVP to immunogenic metabolites, as well as by increased phagocytosis of the immunogenic metabolites (innate immunity).

Of note, the release of Th1 cytokines was more immediate with LPS than with NVP treatment. Specifically, during the chronic phase, IL-2 concentrations continued to rise in the NVP only group while it remained static in the LPS + NVP group. Increased IL-2 with lymphocytosis in presence a normal liver in the NVP group may, on one hand, imply a protective mechanism whereby IL-2 maintained the immunological mechanisms responsible for mopping up the antigenic protein adducts that are continuously formed with continued NVP dosing. This also explains the slow onset of IL-2 stimulation as due to effective antioxidant mechanisms at the start of treatment that neutralize most of the toxic or reactive metabolites, but with continued dosing, these antioxidant mechanisms become depleted or less effective, leading to a progressive buildup of the antigenic metabolites, which then triggers the immune system to activate the destructive immune pathway (release IL-2). On the other hand, since IL-2 is a Th1 cytokine that mediates the destructive immune response, the persistent increase in IL-2 with lymphocytosis would pause a risk to development of hepatotoxicity. Indeed, this may explain why patients with high CD4 are predisposed to development of NVP-induced hepatotoxicity. In effect, there is always a risk of hepatotoxicity on chronic dosing with NVP. Conversely, the IL-2 mechanism was not required in the LPS + NVP group because there is reduced or no metabolic activation of NVP.

The findings of this study have a lot in common with those reported for NVP induced skin reactions. In the current study, NVP 200 mg/kg was used after lower doses led to undetectable NVP concentrations by 24 hours. This concurred with earlier reports where, at the same dose of NVP 150 mg/kg, there were less skin lesions (20%) and lower NVP concentrations in SD rats compared to 100% in brown norwegian (BN) rats [7]. In another report, NVP-induced skin reactions were prevented by inhibition of cytochrome P450 with aminobenzotriazole [33, 34]. Furthermore, NVP-induced skin reactions were prevented by use of the immunosuppressant drug cyclosporine, a known inhibitor of Il-2 production [8]. In effect, the NVP-induced liver injury observed in the current study is most probable similar to that observed in humans, excluding the idiosyncratic hepatotoxicity because it is difficult to prove [34].

**Figure 5:**
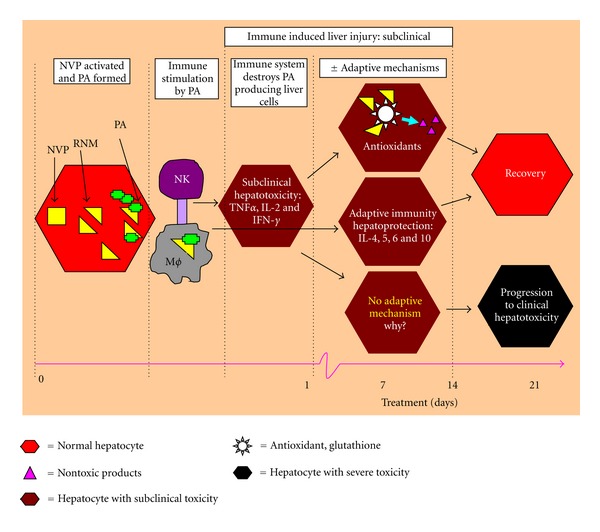
An illustration of the possible subclinical immune-pathological changes in the liver during treatment with nevirapine in rats over the 21 days. NVP: nevirapine, RNM: reactive nevirapine metabolites, PA: protein adducts, NK: natural killer cells, and MØ: macrophage.

This essence of this study is summarized in Figure 5. It shows the underlying principle by which this animal model was used to demonstrate the early immunological responses and resultant pathological changes in the liver during NVP therapy over 21 days. It is proposed here that NVP was metabolized to reactive metabolites which formed antigenic protein adducts, and these led to activation of the destructive immune pathway (proinflammatory Th1 cytokines) in an attempt to get rid of the antigenic protein adducts. Unfortunately, the activated immune system also attacked the liver cells expressing the antigenic adducts, leading to a subclinical liver injury. However, this was counteracted by an opposing (protective) immune response of adaptive mechanisms which, among others, includes the anti-inflammatory Th2 cytokines (IL-4, IL-5, IL-6, and IL-10), leading to immune tolerance. Along with these, antioxidant mechanisms helped to quench the toxic metabolites to prevent formation of protein adducts. The two mechanisms led to recovery of the subclinical liver injury. Unfortunately, in a rare invent, when the two adaptive mechanisms fail to counter the Th1 response, the subclinical liver injury progresses to overt clinical hepatotoxicity. Of note, progression to clinical hepatotoxicity was not tested here because it was demonstrated in our previous report as explained in the first paragraph of this discussion [21].

In the clinical setting, it is believed that most patients overcome this early subclinical liver injury as a result of the adaptive and antioxidant mechanisms, but in a small group of patients these mechanisms fail leading to overt clinical hepatotoxicity in the early phase of treatment (2–8 weeks) [1]. Specifically, symptomatic hepatic events (regardless of severity) occur in 4% of patients on nevirapine treatment within the first six weeks of treatment and is more common in HIV + female patients with a CD4 count > 250 cells/mm^3^ and HIV + male patients with a CD4 count > 400 cells/mm^3^ [1, 2]. Thus, increased CD4 count is considered as a predisposing factor to NVP-induced liver injury and IL-2, a cytokine that was stimulated by NVP therapy, has been shown to increase CD4 count mainly by expanding CD4 cells and prolonging their half-lives [35]. The latter confirms that increased IL-2 predisposes to development of NVP-induced hepatotoxicity, and that although the occurrence of hepatotoxicity is less frequent after 8 weeks of NVP therapy, it can occur at any time of NVP treatment [1]. This animal model appeals to that small group of patients who progress to overt clinical hepatotoxicity. Since LPS completely prevented NVP-induced subclinical liver injury, and therefore the risk of NVP hepatotoxicity, this implies that understanding the mechanism involved may give a clue to development of an ultimate therapeutic agent, and this animal model is a vital research tool by which to investigate this aspect further.

This study also highlights the fact that changes in the immune system after single-dose administration of NVP should never be used to make conclusions on the effects of drugs on the immune system during chronic drug therapy. Specifically in this case, stimulation of IL-2 production by NVP could not have been confirmed without chronic dosing. Also, it was recognized that whereas a thorough understanding of the relationship between cytokines and NVP-induced subclinical liver injury requires measuring the hepatic concentrations of the respective cytokines, this was not done in this study. However, this study should be taken in the same light as other studies that used the same experimental design, that is, diclofenac [9], ranitidine [14], and trovafloxacin [15], with a hope that these results will foster further research in this field.

In conclusion, it has been demonstrated that NVP is a slow onset immune stimulant, NVP-induced subclinical liver injury was associated with immune stimulation by NVP itself, and that LPS prevents NVP-induced subclinical liver injury. These observations confirm that the immune system in involved in NVP toxicity, and that manipulation of the immune system may be one way to prevent NVP-induced hepatotoxicity.

## Figures and Tables

**Table 1:** Average weights (mean ± SD) and liver function test of each of the four groups (*n* = 5) in the acute phase, at 6 and 24 hours after dosing.

Group (*n* = 5)	Weight (g)	Liver function (units/L)		
		ALP	ALT	AST
Untreated				
0 hours	392 ± 13	262.5 ± 11	67.8 ± 24	169.3 ± 107
LPS + S				
6 hours	302 ± 22	214.2 ± 32	83.2 ± 28	161.0 ± 23
24 hours	286 ± 20	274.8 ± 35	30.4 ± 3	131.4 ± 57
S + NVP				
6 hours	372 ± 26	268.0 ± 49	53.2 ± 6	31.4 ± 65
24 hours	367 ± 32	271.6 ± 38	67.2 ± 8	135.0 ± 28
LPS + NVP				
6 hours	379 ± 18	175.6 ± 32	61.0 ± 16	134.2 ± 57
24 hours	385 ± 8	205.8 ± 24	31.0 ± 8	106.6 ± 20

Key: NVP: nevirapine, LPS: lipopolysaccharide, S: saline, ALP: alkaline phosphatase, ALT: alanine aminotransferase, and AST: aspartate aminotransferase.

**Table 2:** Average weights (mean ± SD) and liver function tests of rats (*n* = 5) in the chronic phase after 7, 14, and 21 days of dosing.

Group	Weight (g)			Liver function tests (units/L)		
(*n* = 5)	Before Rx	After Rx	Change	ALP	ALT	AST
Untreated						
7 days	392 ± 13	404 ± 12	12 ± 4	262.5 ± 11	**67.8** ± 24	169.3 ± 107
14 days	399 ± 14	419 ± 21	21 ± 10	273.8 ± 57	**55.4** ± 5	115.8 ± 10
21 days	376 ± 24	397 ± 11	22 ± 4	285.0 ± 34	**57.8** ± 6	120.8 ± 15
(S + NVP)						
7 days	264 ± 5	274 ± 11	10 ± 7	194.8 ± 31	**58.5** ± 10	139.0 ± 58
14 days	260 ± 8	278 ± 32	17 ± 24	208.7 ± 31	**75.3** ± 6	126.0 ± 25
21 days	279 ± 14	310 ± 4	31 ± 11	152.0 ± 64	**49.0** ± 7	112.3 ± 12
(LPS + NVP)						
7 days	271 ± 5	256 ± 13	−11 ± 9	403.3 ± 28	**147.**0 ± 13	427.0 ± 40
14 days	269 ± 7	277 ± 8	8.0 ± 4	150.6 ± 11	**52.0** ± 12	111.8 ± 26
21 days	272 ± 9	295 ± 13	23 ± 9	165.0 ± 12	**33.8** ± 7	89.8 ± 14

Key: Rx: treatment (Rx refers to test groups); S: saline; NVP: nevirapine; LPS: lipopolysaccharide; ALP: alkaline phosphatase; ALT: alanine aminotransferase; AST: aspartate aminotransferase.

**Table 3:** Full blood count data (mean ± SD) for groups of animals (*n* = 5) after 21 days of dosing with saline and nevirapine (S + NVP) and lipopolysaccharide and nevirapine (LPS + NVP).

	S + NVP	LPS + NVP	Reference range
Red blood cells			
Red cell count	8.91 ± 0.4	9.08 ± 0.1	4.5–5.9 × 10^12^/L
Haemoglobin	16.70 ± 0.7	16.93 ± 0.5	13.0–18.0 g/dL
Haematocrit	0.74 ± 0.0	0.73 ± 0.0	0.40–0.50 L/L
MCV	83.33 ± 1.2	83.0 ± 1.0	81–100 fL
MCH	19 ± 0.0	19.0 ± 0.5	28–35 pg
MCHC	22.3 ± 0.6	22.7 ± 0.6	32–36 g/dL
White blood cells			
White cell count	10.90 ± 0.5	15.33 ± 2.1	4.0–11.0 × 10^9^/L
Neutrophils	2.22 ± 0.6	8.89 ± 1.3	2.0–7.50 × 10^9^/L
Lymphocytes	7.95 ± 1.4	5.33 ± 1.1	1.0–4.00 × 10^9^/L
Monocytes	0.59 ± 0.1	0.74 ± 0.5	0.0–0.95 × 10^9^/L
Eosinophils	0.10 ± 0.0	0.57 ± 0.5	0.0–0.40 × 10^9^/L
Basophils	0.10 ± 0.0	0.00 ± 0.0	0.0–0.10 × 10^9^/L
Others			
Platelet count	975 ± 184	601 ± 85.4	140–420 × 10^9^/L

Key: S: saline, NVP: nevirapine, LPS: lipopolysaccharide, MCV: mean corpuscular volume, MCH: mean corpuscular haemoglobin, and MCHC: mean corpuscular haemoglobin concentration.
